# Refined Analysis of Older eHealth Users From an Agency Perspective: Quantitative Telephone Interview Study

**DOI:** 10.2196/40004

**Published:** 2023-04-26

**Authors:** Esther Brainin, Efrat Neter

**Affiliations:** 1 Ruppin Academic Center Department of Behavioral Sciences Faculty of Community and Social Sciences Emek Hefer District Israel

**Keywords:** eHealth, health, internet, structuration theory, agency, digital divide, age, gender, education, information, health condition, self-rated health, SRH, health care services, surrogate, older adults, users, patient, Giddens, Archer, Bourdieu, capital

## Abstract

**Background:**

Most studies on the eHealth divide among older people have compared users to nonusers and found that age, gender, and education were associated with eHealth misuse. They assumed that these characteristics were structural barriers to eHealth adoption. Furthermore, eHealth practices have been examined in a narrow and incomplete way, and the studies disagree about the association between health conditions and eHealth use. Using a more dynamic theoretical lens, we investigated the potential motivations driving older adults’ agential adoption of eHealth practices despite their advanced age.

**Objective:**

This study aimed to obtain a complete and detailed description of eHealth uses among older adults; examine whether demographic characteristics such as age, gender, and education (previously related to eHealth misuse) are still associated with the various eHealth clusters; and determine whether contextual factors such as changes in the health condition of older eHealth users or their loved ones are associated with older adult eHealth use.

**Methods:**

We conducted a 30-minute telephone interview with a representative sample of 442 Israeli adults (aged ≥50 years) with a sampling error of 2.04%. The interviews were conducted in Hebrew, Arabic, and Russian. Using factor analysis with 21 eHealth use questions, we identified 4 eHealth clusters: instrumental and administrative information seeking, information sharing, seeking information from peers, and web-based self-tracking. In addition to age, gender, education, internet experience, frequency of internet use, perceived eHealth literacy, and self-rated health, we asked respondents to indicate how much they had used offline health services because of a health crisis in the past year.

**Results:**

We found differences in the number of older eHealth users in the various clusters. They used instrumental and administrative information (420/442, 95%) and obtained information from peers (348/442, 78.7%) the most; followed by web-based self-tracking related to health issues (305/442, 69%), and only a few (52/442, 11.3%) uploaded and shared health information on the web. When controlling for personal attributes, age, gender, and education were no longer predictors of eHealth use, nor was a chronic ailment. Instead, internet experience, frequency of internet use, and perceived eHealth literacy were associated with 3 eHealth clusters. Looking for health information for family and friends predicted all 4 eHealth clusters.

**Conclusions:**

Many older adults can overcome structural barriers such as age, gender, and education. The change in their or their loved ones’ circumstances encouraged them to make deliberate efforts to embrace the new practices expected from today’s patients. Seeking health information for family and friends and dealing with unexpected health crises motivates them to use eHealth. We suggest that health professionals ignore their tendency to label older people as nonusers and encourage them to benefit from using eHealth and overcome stereotypical ways of perceiving these patients.

## Introduction

### Background

Age is a well-known predictor of the eHealth divide [1-4]. However, there is mounting evidence that older people, particularly those in high-income countries, are among the fastest-growing users of eHealth [5-7]. Our study sought to portray a diverse range of eHealth uses among older adults; explain what trajectories might have led to this shift in eHealth use; and find out whether age, gender, and education (previously predicting non-eHealth use) will be associated with the amount of eHealth use among older adults. To that end, we proposed to rely on a different theoretical lens than the one used by most previous studies on the gray eHealth divide and conduct a study only among older eHealth users. Our study filled these gaps by addressing four objectives as follows: (1) to obtain a comprehensive and detailed description of eHealth uses among older adults and classify them into clusters; (2) to examine whether demographic characteristics such as age, gender, and education (previously associated with eHealth misuse) continue to be associated with the different eHealth clusters; and (3) and (4) to find out which contextual factors, such as older eHealth users’ and their loved ones’ changing health conditions, are associated with their use of eHealth in the different clusters.

### Study Novelty and Knowledge Gaps

Most studies on the *eHealth divide* have explained why there are differences between older people who use eHealth and those who do not. However, they have ignored the possibility of a change in this practice trajectory. The Bourdieusian theoretical framework has been used in many studies to explain this divide [2,8-10]. However, this line of explanation has been criticized as being too deterministic and rigid [11,12]. We proposed that the structuration theory by Giddens [13,14] can explain this transition as he argues that social structure forces (such as belonging to the older age group [15] and being reluctant to use the internet) can be changed through a process of internal deliberation that people (agents) are doing when they face changes in their lives’ contexts. They have the freedom to modify their own goals in relation to their changing context, choose projects, and translate them into new practices [16]. Bourdieu [8] and Giddens [13,14] differ in how they view social actors’ conscious intentions. For Giddens [13,14], actors are reflexive; they can reflect on their actions and identities and act accordingly [17]. He defines *agency* as the ability and deliberateness to achieve goals through a conscious reflection on one’s habitus [18-20]. In his view, context matters as it sets social expectations; makes agents reflect on their daily behavior; and may encourage them to modify their goals and embrace social expectations, especially in unforeseen situations [21]. Although this study is neither causal nor longitudinal, we explained the 2 things that have changed in the context of health service use in recent years: the social expectations regarding a patient’s role [22] and pervasive internet access. Laypeople have gained technical and medical knowledge, skills, and expertise through the media and new technologies, resulting in *lay reskilling* encouraged by the availability of electronic information, policy makers, and institutions [23,24]. It reconstructed the new patient’s identity [25] and set new social expectations for powerful autonomy. These changes were intertwined with internet penetration, suggesting that the first digital divide related to access has been closed [26], and thus, obtaining health information on the web became a proactive behavioral adaptation to later-life health challenges [27-29], representing an agentic approach to positive health behaviors and self-care [30]. To study the use of eHealth as an agentic behavior among older adults, we need a much more detailed and nuanced elaboration of the term *eHealth* as, although it is nearly 20 years old, we found a narrow operationalization and little consistency in how eHealth was defined and measured in the scientific literature on older adults’ eHealth use.

### Defining eHealth

We looked at quantitative studies published in the last 10 years that examined eHealth use among middle-aged and older adults using large, population-representative survey data sets (Multimedia Appendix 1 [1,3-6,31-38]). There were 77% (10/13) of the studies that examined eHealth use as a predicted variable and 23% (3/13) of the studies that examined it as a predictor variable. Most of the studies (9/13, 69%) in Multimedia Appendix 1 were conducted in the United States, with the rest being conducted in Poland, Israel, and Sweden. Most studies (10/13, 77%) measured eHealth as the predicted variable to identify its predictors, which explain its association with offline health service use [6], better self-care and users’ empowerment [4], and medical satisfaction [31]. Some studies (2/13, 15%) defined eHealth use as “health information technology” [6,32], whereas others (1/13, 8%) defined it as “using social media for health-related activities” [33]. With some similarities, each study used 1 to 5 questions about eHealth use. In total, 77% (10/13) of the studies examined whether respondents looked on the web for general health information (about health or illness) [5]. The second most frequently asked eHealth use question (6/13, 46%) was whether respondents had used the internet to schedule a medical appointment [32], deal with health insurance [3], or refill a prescription [34] or whether they had contacted their medical provider directly [1]. Respondents were asked if they had sought health information [3], started or joined a health-related support group [35], or used chat groups to learn about health topics (3/13, 23%). Only 8% (1/13) of the studies examined whether participants kept a web-based diary or blog [35]. To conclude, recent research on eHealth use among middle-aged and older adults has revealed an incomplete and limited picture of the potential eHealth use. Thus, the first and second research questions of our study filled these gaps. First, we obtained a comprehensive and detailed description of the various eHealth uses among older adults. Second, we determined whether demographic characteristics such as age, gender, and education (previously related to non-eHealth users) continue to be associated with the use of eHealth in the different clusters. Notwithstanding, apart from the rich eHealth options available to users, there is another contextual factor that might facilitate nonusers’ transition to become users—users’ or their loved ones’ unexpected changes in health condition.

### Measuring Health Condition Change Through Health Care Use

There was conflicting evidence related to the association between health status and eHealth use. People with chronic conditions, disabilities, or low self-rated health (SRH) were most likely to seek out and act on health news and information [39,40]. This was true for older participants as well [1,32,36,40]. In contrast, other studies found that web-based health information seekers were mostly healthy [2,41,42]. A study on older adults found no relationship between eHealth use and SRH [36]. A recent longitudinal, 2-cycle study explored the relationships among chronic conditions (representing health status), eHealth use, and health care use [6]. Both study cycles showed an association between eHealth use and physician visits (including emergency room or clinic visits); however, the association was stronger in the first cycle, when they were coterminous. The use of eHealth information was also linked to fewer physician visits among participants with 1 in 5 chronic conditions (diabetes). Wicks et al [43] found a negative relationship between eHealth use and physician visits. In total, 2 recent studies examined eHealth use before and after visiting a health care provider and health care use, which was not measured using physician visits. According to these studies, eHealth empowers patients and improves self-care and health perceptions [4,31]. In this study, we assumed that eHealth use can have a more crucial effect among those who experience a change in their own or a loved one’s health [4], especially among the older population. As the focus of this study was on cases in which a change in one’s own or a loved one’s health condition served as a potential stressor [44], which facilitates the emergence of active eHealth users, it was important to conceptualize and measure changes in health status. The aforementioned studies used different health measures. Some studies used the total number of chronic illnesses diagnosed (followed by a list of the most common chronic illnesses), whereas others asked respondents to self-report activity restrictions and memory issues, among other things. It was suggested that the SRH responses may be sensitive to the wording of both the question and the response options [45]. It was not linked to eHealth after demographic variables were considered [33], and its measure may reflect a general estimation of one’s health condition related to a given period, disregarding any coincidental or random episode of change in one’s health condition that occurred during the research period but was resolved. Thus, although very popular, SRH may not reflect a recent health change. Chronic illness is an irreversible medical condition that may require lifelong adaptation and management, so treatment becomes part of one’s daily routine. To capture major changes in health conditions during a certain period, we proposed using health care use as a proxy. In most cases, such a change requires using the multitiered health care system, such as seeing a general practitioner and a specialist or needing emergency services or hospitalization. Patients with major health changes quickly use the entire system’s layered structures. Given that physicians cannot always satisfy patients’ information needs [41] and that patient autonomy in making treatment decisions is encouraged, turning to eHealth resources may be a stress-buffering agent [44]. It transforms patients from passive health consumers into proactive health producers with knowledge [44]. To support our third research question, we suggested that recent health care use among old eHealth users can provide a thorough and detailed measure of health status change when studying eHealth uses and their relationship to changes in contextual factors of eHealth users that might turn them into active agents who use eHealth intensively.

### Knowing Means Participating—Older Adults as Health Information Surrogates

People search for eHealth not only for themselves but also on behalf of others without being asked to do so, often initiating behavior change or influencing health-related decisions [46,47]. Lay information intermediaries, or health information surrogates (HISs), seek information in a self-generated (ie, internally motivated), nonprofessional, or informal capacity, anticipating another person’s needs. These agents, also called *hidden patients*, are proxy searchers with purposeful, problem-driven behavior [48,49]. A possible motivation for this behavior is another contextual change for the seeker—a loved one’s health change. In such situations, people seek health information on behalf of their family, friends, or colleagues as caregivers or significant others, sometimes at higher rates than they do for themselves [42]. Individuals who seek others’ health-related information can promote better transmission of information and social support [50]. Cutrona et al [46] found that two-thirds (66.6%) of American respondents reported being HISs between 2011 and 2012. Surrogate seekers reported more eHealth activities requiring user-generated content, such as emailing health care providers, visiting social networking sites to read and share medical topics, and joining online health support groups. This number is higher than that of a 2012 Pew Internet Poll (54%) [51]. Europe had similar results. Data from the Flash Eurobarometer on 28 European Union member states showed that 61% of respondents searched for health-related information on behalf of someone else [52]. Middle-aged Europeans were the most likely to report being HISs, whereas the youngest and oldest were the least likely to do so. Despite these findings, the study suggests exploring HISs among older adults aged ≥50 years because of their varied family roles and social engagements. These engagements are part of the eHealth users’ context. The group aged 50 to 64 years may be similar to the European middle-aged group with the most HISs. For users aged ≥65 years, this measure is also important as their generational identity is changing because of increased life expectancy and technology exposure. Most people will spend a longer period of their lives in a 3- to 4-generation family. This age group has considerable demographic and social weight as it can devote more time to intergenerational and friendship help [53-55]. Although studies have explored respondents’ HIS behavior, most of them used general yes-or-no questions without specifying what kind of information they were looking for. Our study filled the gap in the literature by investigating which eHealth practices older adults use that are associated with their HIS behavior.

## Methods

### Overview

This study used data collected through telephone interviews using a national random-digital dial-telephone household survey of Israeli adults (aged ≥50 years). The interviews lasted between 25 and 30 minutes. The interviews were conducted in Hebrew, Arabic, and Russian by professional interviewers who went through a special training session to familiarize themselves with the questionnaire’s terminology. After a short introduction, each interviewee was asked whether they agreed to participate in the survey. Those who agreed were then interviewed. The interviewers conducted the telephone survey using computer-assisted telephone interviewing software.

### Ethics Approval

This study was approved by the institutional review board of Ruppin Academic Center (2012-1-6). All respondents expressed consent before their participation. Respondents’ anonymity was assured, and they were not asked for any identifying information during the phone survey.

### Sample

Calls were placed to 1288 representative residential households, of which 128 (9.94%) were not relevant (eg, disconnected, business, or fax numbers). Of the remaining 1160 households, 603 agreed to be interviewed, representing a 51.98% response rate and a sampling error of 2.04%. The participation criterion for the first survey phase was the respondents’ age (≥50 years). In this phase, there were 34.5% (208/603) of participants who did not use the internet at all, 28.5% (172/603) of participants who used the internet but not for health purposes, and 37% (223/603) of participants who used the internet for health purposes—the eHealth users. All 3 groups were interviewed. To focus on older eHealth users only and portray the extent and scope of eHealth activities on a larger sample, the data collection process followed a second phase using the same methodology as the one used in phase 1 except that the following 2 participation criteria were used: respondents’ age (≥50 years) and respondents’ use of the internet for health-related purposes. In phase 2, only participants who met the 2 criteria were interviewed. As a result, of the 1139 representative residential households that were contacted, an additional 219 (19.23%) eHealth participants were surveyed, resulting in a sample of 442 eHealth participants.

### Design

The explained measures of eHealth activities included 4 clusters: instrumental and administrative information seeking, information sharing, seeking information from peers, and self-tracking. The explanatory variables included demographic variables such as age, gender, education, and marital status; health-related variables such as chronic ailments, seeking information for family and friends, and offline health care use; and internet use frequency, experience, and skills.

### Measures

#### Clusters of eHealth Activities

eHealth activities were explored using 21 questions that examined participants’ detailed engagement in web-based health-related activities. A total of 10 items were measured using a 5-point frequency scale (1=never; 5=several times a week) in response to the following question: “How often did you use the Internet for the following health issues?” A total of 11 items were measured using a dichotomous yes-or-no scale in response to the following question: “Did you engage in the following online health-related activities in the past twelve months?” Subsequently, using principal component analysis with varimax rotation, eHealth activities were clustered into 4 categories, which explained 33.9% of the variance. The 4 clusters of eHealth activities that emerged were instrumental and administrative information seeking (10 items; mean 1.53, SD 0.42; Cronbach α=.73; highest-loading item: “Sought information about long term care for an elderly or disabled person”), information sharing (3 items; mean 1.06, SD 0.19; Cronbach α=.74; highest-loading item: “Posted a review web-based of a particular drug or medical treatment”), seeking information from peers (5 items; mean 1.43, SD 0.32; Cronbach α=.68; highest-loading item: “Sought others who might have health concerns similar to mine”), and self-tracking on the web (3 items; mean 1.42, SD 0.36; Cronbach α=.57; highest-loading item: “Tracked my health indicators or symptoms online”).

#### Demographic and Health-Related Variables

Participants were asked to provide their age, gender, education, and marital status. We have consistently reported on gender in the manuscript. The gender variable included 2 values. Offline health care use was measured by asking respondents whether they had experienced a major change in their health condition, seen their general practitioner, seen a specialist, sought emergency room services, or been hospitalized in the past year. Responses were provided using a 3-point response scale (1=no; 2=yes, once or twice; and 3=yes, 3 times or more). The possible response score obtainable for this independent variable was between 5 and 15 (mean 7.86, SD 1.86). The chronic ailments variable was measured using a 3-point response scale (1=no chronic ailment, 2=1 or 2 chronic ailments, and 3=3 or more chronic ailments). Health information seeking for family and friends was measured using a dichotomous yes-or-no scale in response to the following question: “For whom have you looked for health information online in the past year? (Yourself, your spouse, children, parents, relatives, friends).” The scale was scored by adding the answers, resulting in a score ranging from 6 to 12 (mean 8.13, SD 1.53).

#### Internet Use Frequency, Experience, and Skills

Participants’ internet experience was measured by asking respondents to report the number of years since they first began surfing the web (mean 11.02, SD 5.93); the frequency of internet use was measured by asking respondents how often they generally used the internet. Responses were provided using a 7-point scale (1=very seldom; 7=every day, all day; mean 5.71, SD 1.02). eHealth literacy was measured using the eHEALS tool [56]. The scale comprises 8 items evaluated on a 5-point Likert scale (1=strongly disagree; 5=strongly agree; mean 3.1, SD 0.83; Cronbach α=.90; sample item: “I know how to find helpful health-related resources on the Internet”). The scale was previously translated into Hebrew [10].

### Data Analysis

First, the sample’s demographic and background characteristics and the eHealth cluster activities were described using descriptive statistics. Second, Pearson correlations between the 4 eHealth clusters of activities and all other variables were computed. Third, hierarchical multiple linear regression analyses were carried out on the 4 eHealth clusters of activities. Age, gender, education, and marital status were entered in the first step, and health-related variables, internet use frequency, experience, and skills were entered in the second step. Each step presents its contribution to the explained variance. The analyses were conducted using SPSS Statistics (version 23; IBM Corp).

## Results

### Sample Demographic and Background Characteristics

The characteristics of the participants are presented in Table 1. The sample comprised 63.1% (279/442) women and 95.2% (421/442) Jewish participants. Participants’ age ranged from 50 to 87 years with a mean age of 61.05 (SD 8.23) years. The mean age of the men participants was considerably higher than that of women participants (mean 62.2, SD 8.85 vs mean 60.38, SD 7.77, respectively; *F*_1,439_=5.07; *P*=.03). Approximately half (225/442, 50.9%) of the sample had postsecondary education, and 74.4% (329/442) were married. A total of 38% (168/442) of the participants reported one or more chronic ailments.

**Table 1:** Demographic profile of older eHealth users (n=442).

Characteristic			Participants, n (%)
**Gender**			
	Men	163 (36.9)	
	Women	279 (63.1)	
**Age (years)**			
	50-54	116 (26.2)	
	55-59	98 (22.2)	
	60-64	91 (20.6)	
	65-69	65 (14.7)	
	70-74	33 (7.5)	
	75-84	38 (8.6)	
**Education**			
	Secondary education	215 (48.6)	
	Postsecondary and tertiary education	225 (50.9)	
**Marital status**			
	Single, divorced, or widowed	96 (21.7)	
	Married	329 (74.4)	
**Ethnicity**			
	Jewish	421 (95.2)	
	Arabic	21 (4.8)	
**Health condition—chronic ailments**			
	No chronic ailments	262 (59.3)	
	One or more chronic ailments	168 (39)	
**Health condition—change in health condition in the past 12 months**			
	No	214 (48.4)	
	Yes	221 (50)	
**Internet use frequency**			
	Once a week or less	14 (3.2)	
	Several times a week	48 (10.9)	
	Once a day	49 (11.1)	
	Several times a day	261 (59)	
	All day, every day	69 (15.6)	

### Clusters of eHealth Activities

To obtain a comprehensive and detailed description of eHealth practices among older adults, we divided the sample into 6 age groups. As explained in the *Methods* section, different numbers of activities were classified into each of the 4 eHealth clusters. The clusters of eHealth uses and the amount of users in the various age groups are presented in Table 2.

For every cluster of eHealth practices, we calculated the percentage of participants of different age groups, and the mean percentage of all age groups is also displayed. Table 2 shows that, for *instrumental and administrative information seeking*, the practice rate was the highest, ranging from 88% (29/33) for the age group of 70 to 74 years to 97% (95/98) for the age group of 55 to 59 years, with a mean use of 94% (SD 3.20%). Next was the *seeking information from peers* cluster, ranging from 85% (28/33) for the age group of 70 to 74 years to the lowest percentage (27/38, 71%) for the oldest age group of ≥74 years (mean use 79%, SD 4.86%). The third cluster of use was *self-tracking on the web*, ranging from 74% (48/65) for the age group of 65 to 69 years to 63% (62/98) for the age group of 55 to 59 years. The mean use for this cluster was 69% (SD 4.83%). The least frequent uses of eHealth were in the *information-sharing practices* cluster, ranging from 13.8% (16/116) for the youngest age groups to 3% (1/38) for the oldest. Only a mean of 11% (SD 4.87%) of participants used eHealth in this cluster. We can see that there are few differences in the percentage of users between age groups within each cluster. However, there are significant differences in the percentage of users between clusters.

**Table 2:** Clusters of eHealth practices by different age groups.

Cluster	Age group (years), n (%)							Value (%), mean (SD)
	50-54 (n=116)	55-59 (n=98)	60-64 (n=91)	65-69 (n=65)	70-74 (n=33)	≥74 (n=38)		
Instrumental and administrative information	n (95)	95 (97)	n (96)	n (95)	29 (88)	n (95)	94 (3.20)	
Seeking information from peers	n (78)	n (80)	n (77)	n (83)	28 (85)	27 (71)	79 (4.86)	
Self-tracking on the web	n (66)	62 (63)	n (72)	48 (74)	n (73)	n (66)	69 (4.83)	
Information sharing	16 (14)	n (14)	n (10)	n (8)	n (15)	1 (3)	11 (4.87)	

### Pearson Correlations Between the 4 eHealth Clusters and All Other Variables

The intercorrelations between the 4 clusters of activities and all independent variables are displayed in Table 3. We can see from the table that the 4 eHealth clusters are significantly associated with each other at moderate to high levels so that the more one engages in one cluster of activity, the more likely it is that one engages also in the other activities. Instrumental and administrative information seeking had significant positive correlations with the other 3 eHealth activities: information sharing (*r*=.31; *P*<.001), seeking information from peers, (*r*=.38; *P*<.001) and self-tracking (*r*=.48; *P*<.001). The correlations between information sharing and seeking information from peers and self-tracking activities were statistically significant but at a lower value (*r*=0.21 and *P*<.001 vs *r*=0.16 and *P*<.001, respectively). Seeking information from peers was also correlated with self-tracking at a moderate level (*r*=0.37; *P*<.001). Despite the large scale of the sample age, there were no significant correlations between age and the 4 eHealth activities, nor were they associated with gender, except for seeking information from peers. Women were found to use information from peers more than men (*r*=0.13; *P*=.01). Education was significantly correlated with 3 eHealth activities except for information sharing. Married respondents were significantly more likely to use information from peers (*r*=0.13; *P*=.01). Only 2 of the 3 health-related conditions were significantly correlated with eHealth activities such that health care use and health information seeking for family and friends were significantly correlated with the 4 clusters of eHealth activities and chronic ailments was not. The correlations for health care use were *r*=0.13 (*P*=.01) with instrumental information seeking, *r*=0.11 (*P*=.05) for information sharing, *r*=0.21 (*P*<.001) for seeking information from peers, and *r*=0.17 (*P*<.001) for self-tracking. The highest correlation of health information seeking for family and friends was with seeking information from peers (*r*=0.45; *P*<.001), followed by instrumental information seeking (*r*=0.39; *P*<.001), self-tracking (*r*=0.27; *P*<.001), and information sharing (*r*=0.12; *P*=.01). Offline health care use (which served as a proxy for health condition change) was also statistically significant and positive for all 4 eHealth activities but at a lower level. The highest correlation was between offline health care use and seeking information from peers (*r*=0.21; *P*<.001), followed by self-tracking activities (*r*=0.17; *P*<.001), instrumental information seeking (*r*=0.13; *P*=.01), and information sharing (*r*=0.11, *P*=.05).

The 3 variables related to internet use frequency, experience, and eHealth literacy were positively correlated with eHealth activities except for information sharing. eHealth literacy had the highest correlation with seeking information from peers (*r*=0.43; *P*<.001), followed by instrumental information seeking (*r*=0.40; *P*<.001), self-tracking activities (*r*=0.26; *P*<.001), and information sharing (*r*=0.10; *P*=.05). Internet use frequency was positively associated with instrumental information seeking (*r*=0.24; *P*<.001), followed by self-tracking activities (*r*=0.18; *P*<.001) and seeking information from peers (*r*=0.16; *P*<.001).

**Table 3:** Correlation analysis (Pearson r and 2-tailed *P* value) between all the study variables.

Variable		1	2	3	4	5	6	7	8	9	10	11	12	13
**1. Instrumental information seeking**														
	*r*	1	0.31	0.48	0.38	−0.07	0.16	0.06	0.06	0.39	0.13	0.17	0.24	0.40
	*P* value	—^a^	<.001	<.001	<.001	.12	<.001	.18	.25	<.001	.005	<.001	<.001	<.001
**2. Information sharing**														
	*r*	0.31	1	0.21	0.16	−0.05	−0.01	−0.05	−0.03	0.12	0.11	0.05	0.08	0.10
	*P* value	<.001	—	<.001	<.001	.31	.85	.34	.50	.01	.03	.34	.10	.03
**3. Seeking information from peers**														
	*r*	0.48	0.21	1	0.37	−0.07	0.10	0.13	0.07	0.45	0.22	0.13	0.16	0.43
	*P* value	<.001	<.001	—	<.001	.13	.03	.006	.14	<.001	<.001	.007	<.001	<.001
**4. Web-based self-tracking**														
	*r*	0.38	0.16	0.37	1	0.02	0.18	−0.04	0.09	0.27	0.17	0.17	0.18	0.26
	*P* value	<.001	<.001	<.001	—	.70	<.001	.48	.052	<.001	<.001	<.001	<.001	<.001
**5. Age**														
	*r*	−0.07	−0.05	−0.07	0.02	1	−0.02	−0.11	0.24	−0.15	0.10	−0.16	−0.16	−0.18
	*P* value	.12	.31	.13	.70	—	.65	.03	<.001	<.001	.04	<.001	<.001	<.001
**6. Education**														
	*r*	0.16	−0.01	0.10	0.18	−0.02	1	0.01	−0.03	0.07	0.01	0.30	0.30	0.27
	*P* value	<.001	.85	.03	<.001	.65	—	.79	.57	.15	.91	<.001	<.001	<.001
**7. Gender**														
	*r*	0.06	−0.05	0.13	−0.04	−0.11	0.01	1	−0.02	0.17	0.08	−0.04	−0.04	0.06
	*P* value	.18	.34	.006	.48	.03	.79	—	.72	<.001	.09	.38	.30	.24
**8. Chronic ailments**														
	*r*	0.06	−0.03	0.07	0.09	0.24	−0.03	−0.02	1	−0.01	0.41	−0.02	−0.08	−0.03
	*P* value	.25	.50	.14	.052	<.001	.57	.72	—	.85	<.001	.74	.09	.51
**9. Information seeking for family and friends**														
	*r*	0.39	0.12	0.45	0.27	−0.15	0.07	0.17	−0.01	1	0.10	0.13	0.21	0.37
	*P* value	<.001	.01	<.001	<.001	<.001	.15	<.001	.85	—	.03	.005	<.001	<.001
**10. Offline health care use**														
	*r*	0.13	0.11	0.22	0.17	0.10	0.01	0.08	0.41	0.10	1	0.01	−0.06	0.06
	*P* value	.005	.03	<.001	<.001	.04	.91	.09	<.001	.03	—	.80	.20	.23
**11. Internet experience**														
	*r*	0.17	0.05	0.13	0.17	−0.16	0.30	−0.04	−0.02	0.13	0.01	1	0.28	0.34
	*P* value	<.001	.34	.007	<.001	<.001	<.001	.38	.74	.005	.80	—	<.001	<.001
**12. Internet use frequency**														
	*r*	0.24	0.08	0.16	0.18	−0.16	0.30	−0.04	−0.08	0.21	−0.06	0.28	1	0.27
	*P* value	<.001	.10	<.001	<.001	<.001	<.001	.30	.09	<.001	.20	<.001	—	<.001
**13. eHealth literacy**														
	*r*	0.40	0.10	0.43	0.26	−0.18	0.27	0.06	−0.03	0.37	0.06	0.34	0.27	1
	*P* value	<.001	.03	<.001	<.001	<.001	<.001	.24	.51	<.001	.23	<.001	<.001	—

^a^Not applicable.

### Hierarchical Multiple Linear Regression Analyses on eHealth Clusters of Activities

#### Overview

Regression analysis of eHealth activities explored whether the health-related variables, internet use characteristics, and eHealth activities were associated with the 4 clusters of activities after controlling for the demographic variables age, gender, education, and marital status. The results are presented in Tables 4 and 5.

**Table 4:** Hierarchical regression analysis for variables predicting 2 clusters of web-based health activities (n=405).

Explanatory variables	Instrumental and administrative information seeking^a^		Information sharing (health Web 2.0)^b^	
	B (SE)	β	B (SE)	β
Age	0.001 (0.002)	−.012	0.000 (0.001)	−.01
Education	0.008 (0.014)	.027	−0.009 (0.007)	−.069
Gender (men)	0.002 (0.04)	.002	−0.03 (0.019)	−.073
Marital status (not married)	0.056 (0.05)	.057	0.011 (0.021)	.026
Offline health care use	0.021 (0.01)	.092^c^	0.02 (0.05)	.167^d^
Chronic ailments (none)	0.031 (0.033)	.045	−0.024 (0.016)	−.081
Information seeking for family and friends	0.073 (0.013)	.271^e^	−0.015 (0.006)	.13^d^
Internet experience	0.002 (0.003)	.024	0.001 (0.002)	.032
Internet use frequency	0.037 (0.019)	.093^c^	0.014 (0.009)	.083
eHealth literacy	0.126 (0.025)	.254^e^	0.001 (0.012)	.005

^a^*R*^2^=0.26; *F*_10,395_=14.08 (*P<*.001).

^b^*R*^2^=0.6; *F*_10,394_=2.59 (*P=*.005).

^c^*P<*.05.

^d^*P<*.01.

^e^*P<*.001.

**Table 5:** Hierarchical regression analysis for variables predicting 2 clusters of web-based health activities (n=405).

Explanatory variables	Looking for information from peers (health Web 2.0)^a^			Health-related web-based self-tracking^b^	
	B (SE)	β	B (SE)		β
Age	0.001 (0.002)	.028	0.003 (0.002)		.067
Education	−0.002 (0.010)	−.009	0.021 (0.013)		.081
Gender (men)	0.031 (0.028)	.047	−0.056 (0.036)		−.076
Marital status (not married)	0.112 (0.033)	.147^c^	0.033 (0.041)		.039
Offline health care use	0.030 (0.008)	.174^c^	0.027 (0.010)		.143^d^
Chronic ailments (none)	0.013 (0.024)	.024	0.024 (0.031)		.041
Information seeking for family and friends	0.061 (0.010)	.297^c^	0.047 (0.012)		.206^c^
Internet experience	−0.001 (0.002)	−.012	0.003 (0.003)		.056
Internet use frequency	0.011 (0.014)	.034	0.026 (0.018)		.077
eHealth literacy	0.117 (0.018)	.305^c^	0.041 (0.023)		.098

^a^*R*^2^=0.34; *F*_10,395_=19.91 (*P*<.001).

^b^*R*^2^=0.15; *F*_10,395_=6.91 (*P*<.001).

^c^*P*<.001.

^d^*P*<.01.

#### Instrumental and Administrative Information Seeking Cluster

In Table 4, we see that the first step of the demographic variables predicted only 4% of the *instrumental and administrative information seeking* variance (*F*_4,401_=3.93; *P*=.004). The health-related variables in the second step added 13% to the explained variance (*F*_7,398_=13.86; *P*<.001), and the internet use characteristics in the third step added 7% to the explained variance (*F*_10,395_=14.08; *P*<.001). The regression coefficients reported are those of the third step. Offline health care use (β=.09; *t*_395_=1.91; *P*=.06), health information seeking for family and friends (β=.27; *t*_395_=5.55; *P*<.001), frequency of internet use (β=.09; *t*_395_=1.94; *P*=.05), and eHealth literacy (β=.25; *t*_395_=5.00; *P*<.001) were found to be significantly associated with instrumental and administrative information seeking such that the more participants used offline health care services, looked for health information for family and friends, used the internet frequently, and had high eHealth literacy, the more they sought instrumental and administrative information.

#### Information Sharing Cluster

The first step of the demographic variables predicted only 1% of the *information sharing* variance (*F*_4,400_=0.61; *P*=.65). The health-related variables in the second step added 3% to the explained variance (*F*_7,397_=3.25; *P*=.002), and the internet use characteristics in the third step added 2% to the explained variance (*F*_10,394_=2.59; *P*=.005). The regression coefficients reported are those of the third step. Only offline health care use (β=.17; *t*_394_=3.05; *P*=.002) and health information seeking for family and friends (β=.13; *t*_394_=2.32; *P*=.02) were found to be significantly associated with information sharing such that the more participants used offline health care services and looked for health information for family and friends, the more they shared health information on the web.

#### Information Seeking From Peers Cluster

Table 5 shows that the first step of the demographic variables predicted only 5% of the information seeking from peers variance (F4,401=5.35; *P*<.001). The health-related variables in the second step added 21% to the explained variance (F7,398=20.03; *P*<.001), and the internet use characteristics in the third step added 8% to the explained variance (F10,395=19.91; *P*<.001). The regression coefficients reported are those of the third step. Marital status (β=.15; *t*_395_=3.49; *P*<.001), offline health care use (β=.17; *t*_395_=3.80; *P*<.001), health information seeking for family and friends (β=.30; *t*_395_=6.40; *P*<.001), and eHealth literacy (β=.31; *t*_395_=6.32; *P*<.001) were found to be significantly associated with information seeking from peers such that married participants who more frequently used offline health care services, looked for health information for family and friends, and had a higher level of eHealth literacy sought information from peers to a larger extent.

#### Self-tracking Cluster

Finally, in Table 5, we see that the first step of the demographic variables predicted only 3% of the *self-tracking* variance (*F*_4,401_=3.06; *P*=.02). The health-related variables in the second step added 10% to the explained variance (*F*_7,398_=8.38; *P*<.001), and the internet use characteristics in the third step added 2% to the explained variance (*F*_10,395_=6.91; *P*<.001). The regression coefficients reported are those of the third step. Only offline health care use (β=.14; *t*_395_=2.75; *P*=.006) and health information seeking for family and friends (β=.21; *t*_395_=3.92; *P*<.001) were found to be significantly associated with self-tracking such that the more participants used offline health care services and looked for health information for family and friends, the more they performed self-tracking activities.

#### Comparing the 4 Clusters

Instrumental information consumption was more prevalent among participants who had looked for health information for their relatives and friends and used the health care system in the past 12 months, especially among those with high eHealth literacy and frequency of internet use. These nonmaterial capitals helped users navigate the web and consume the information they needed to perform causal interventions for themselves or their loved ones, as opposed to the *information sharing* cluster (using Web 2.0 applications), which was the least prominent cluster of web-based health-related activities. The *seeking information from peers* cluster is a source of human capital as we exchange lay interpersonal knowledge and experience, which was also more prominent among participants who had used the health care system in the past 12 months with greater frequency and had high eHealth literacy. This activity is important as people are more likely to be receptive to information shared by others who are like them [57].

Participants used eHealth in the *web-based self-tracking* cluster to a lesser degree than in the previous 2 clusters, but it was more prominent among participants who had used the health care system in the past 12 months more frequently than their cohorts. The frequency of internet use and high eHealth literacy were found to have a positive impact on self-tracking on the web.

## Discussion

### Principal Descriptive Findings

#### Overview

Most studies on the eHealth divide among older adults in the past 10 years have compared users with nonusers [3,5]. We adopted the structuration theory and focused only on older eHealth users to explore the “full half glass” of internet use for health purposes. We wanted to understand the possible facilitators that encourage older adults’ agential adoption of eHealth practices despite their older age. Such findings are essential as older people, especially in high-income societies, constitute the fastest-growing internet user group.

#### First Research Question

Our descriptive statistics findings provided the answer to our first research question by obtaining a detailed description and portraying the diverse eHealth practices that older adults perform. Although previous studies used only 1 to 5 questions to measure eHealth practices among older adults [32,35], we used 21 questions. We created 4 eHealth clusters following a factor analysis procedure: instrumental and administrative information seeking, seeking information from peers who share the same health situation, web-based self-tracking, and uploading and sharing health information. These clusters served as the multipredicted variables in the multilevel analysis. Older adults used eHealth in the instrumental and administrative information cluster the most (29/33, 88% to 95/98, 97%), followed by 71% (27/38) to 85% (28/33) who obtained information from peers who shared the same health situation. Between 62% (61/98) and 77% (71/92) of the participants used eHealth in the third cluster, web-based self-tracking, whereas the least frequent cluster was uploading and sharing health information on the web (1/38, 3% to 5/33, 15%). The correlation analysis among the 4 eHealth clusters showed a small (*r*_424_=0.16) to medium (*r*_430_=0.48) association among the 4 eHealth clusters, suggesting that they are not entirely distinct.

#### Second Research Question

The second research question asked whether classic personal characteristics such as age, gender, and education were associated with older adults’ use of eHealth in the different clusters. We answered this question by using descriptive, bivariate, and multilevel statistical analyses. As age was the most prevalent predictor of eHealth use or nonuse in most previous studies, we used descriptive statistics to compare each eHealth cluster across 6 age groups. Our comparison revealed that the differences in eHealth use among the 4 clusters were greater than the differences within each eHealth cluster, specifically among the 6 age groups. Age was not a significant predictor of any of the 4 eHealth clusters despite the sample’s large age range. Gender was also insignificant except for the *seeking information from peers* cluster, which had more women than men participants. All clusters except for *information sharing* were significantly correlated with education (albeit at a very low value). In addition to the demographic variables, we checked the correlation between the 4 eHealth clusters and the 3 dimensions of internet use: frequency, experience, and eHealth literacy. We found that these variables were significantly associated with the eHealth clusters except for the *information sharing* cluster. This implies that the more experienced and confident the user is, the more they use most eHealth practices.

#### Third Research Question

To answer the third research question of whether changes in health circumstances are associated with older adults’ use of eHealth in the different clusters, we correlated the 4 eHealth clusters with chronic ailments and recent offline health care use. In contrast to offline health care use, chronic ailments were not associated with any of the eHealth clusters, suggesting that offline health care use expresses a change in the respondents’ health condition in the months that preceded the study. The more respondents used offline health care services, the more they used eHealth in the 4 clusters.

#### Fourth Research Question

The fourth research question was whether looking for health information for family and friends was associated with the 4 eHealth clusters. The literature suggests that, when family and friends experience changes in their health condition, their surrounding circles help them find more information even without being asked to do so. Such behaviors are referred to as HISs. The correlation between the 4 eHealth clusters and the variable “looking for health information online for respondents’ spouses, other family members, and friends” was high and significant.

### Multilevel Analysis Findings

The multivariate analysis revealed more distinct findings. In the first step, we found that the 3 classic background characteristics—age, gender, and education—were no longer significant predictors of the 4 eHealth clusters among eHealth users. Their contribution to the clusters’ explained variance was meager. This finding answers our second research question. In the second step of the multilevel analysis, we found that 2 of the 3 health condition variables were significant predictors of using eHealth in the different clusters. Offline health care use and health information seeking for family and friends can be framed as contextual health situations. We found that these contextual health situations significantly predicted, across a diverse range, all 4 eHealth clusters after controlling for the demographic characteristics. They contributed the most (21%) to the *looking for information from peers* cluster, followed by the *instrumental and administrative information consumption* cluster (15%), the *web-based self-tracking* cluster (10%), and the *information uploading and sharing* cluster (3%). These findings suggest that changes in participants’ and their loved ones’ health conditions are strongly associated with the use of eHealth in 3 of the 4 clusters. The more they used offline health care services in the 12 months preceding the survey and the more they served as HISs, the more they used eHealth in the 3 clusters. These findings are in line with those of the longitudinal study by Shim et al [6]. They found that the use of web-based health information was positively associated with concurrent reports of physician visits but not over 2 years. Controlling for demographic characteristics and health context variables, we found in the third step of the multilevel analysis that internet frequency of use significantly predicted only instrumental and administrative information consumption. Internet experience did not predict any of the 4 eHealth clusters, and eHealth literacy was significantly associated only with instrumental and administrative information consumption and looking for information from peers. These variables contributed an additional 7% to 8% to the explained variance of the use of eHealth in the 2 clusters. We suggest that the low number of respondents who uploaded and shared information is associated with the more advanced skills needed to upload and produce content (Web 2.0). For late adopters, posting a review on the web of a particular drug or medical treatment is more challenging than lurking or retrieving information. In addition, sharing their own experience in a time of health crisis would be questionable. A recent study found that patient collaboration in a physician-patient forum depends on the disease type, time commitment, and incentives [15]. Thus, apart from the 2 contextual health conditions that significantly predicted the use of eHealth in the different clusters, all other variables were insignificant, resulting in an overall poor explained variance.

The findings for research questions 1 and 2 suggest that many older adults can overcome structural barriers such as age, gender, and education. These findings are complemented with the results of research questions 3 and 4, suggesting that the context of our participants’ actions matters. Becoming an HIS by seeking health information for family and friends and using offline health care services in the previous months suggests that an unexpected and unplanned change process occurred in older eHealth users’ or their loved ones’ lives. In a health crisis, patients seek professional support and depend on the structure of the health care system. As the health care system in Israel is public and there is no consideration of the cost of offline services, people do not hesitate to use them. As a result, we believe that increasing the use of public health services can serve as a proxy to measure the worsening of a participant’s health condition. These changes in health condition coincide with new social expectations resulting from the role technology plays in patient-physician relationships, the expectation that patients will take increasing responsibility for their health-related decisions, and easy access to health information and services on the internet. Our study emphasized the need to trace potential change processes as, according to the structuration theory, the context of changes (in the health conditions of participants and their loved ones) matters. This establishes new social expectations that cause people to reflect on their day-to-day conduct. This ongoing reflection process might encourage people to make deliberate efforts to solve the demands of their lives [44] and embrace new practices expected from today’s patients, especially when dealing with unexpected and unplanned contingencies. Although our study was neither longitudinal nor experimental, we suggest that these kinds of changes might explain what drove our participants to become eHealth users despite their old age, especially if they perceived themselves as having high eHealth literacy. The insignificant association between being chronically ill and using eHealth in the 4 clusters posits that monitoring and treating chronic illnesses has become part of daily routines for a long time, in contrast to a sudden change in health condition. We suggest that our findings align with findings that those with a particular chronic health condition using web-based health information were significantly associated with fewer physician visits at both time points of their study [6].

### Limitations, Strengths, and Future Research

Our study is limited primarily because of its cross-sectional methodology. It is neither a longitudinal study nor experimental. Therefore, the associations reported previously might be bidirectional; however, as we asked respondents to describe their actual health circumstances in the previous year, we assume that eHealth use did not cause older eHealth users to feel a sudden change in their health condition followed by extensive use of offline health care services. We also do not believe that people will look for health information on the web for family and friends unless there is a good reason to do so. Despite including representative residential households, our sample limitations arise from our inclusion criteria. Older adults who experienced severe health decline or limitations in physical capacity and more substantial disabilities would not have participated in such a study. As a result, despite the sample’s extensive age range, the participants’ mean age was lower than the mean age of this group of users in the Israeli population. Multiple eHealth practice measures reflect a more fine-grained and detailed description of eHealth uses among older adults and show that, in certain circumstances, such as when confronted with a new health problem, older adults can reflect on their new situation and choose to adopt new practices and use eHealth to deal with offline challenges through an internal deliberation process. For instance, they can consult with peers with little or no effort to obtain advice and support despite their old age. Nevertheless, our study excluded the measurement of mobile technology uses, especially health apps that are very pervasive today, and opens up new opportunities for diagnosing, monitoring, and managing health problems. These findings have implications for potentially expanding the diverse uses of eHealth among older adults. This implies that health professionals should ignore their tendency to label older people as nonusers and encourage them to benefit from using eHealth by overcoming stereotypical ways of perceiving this population.

Future research should continue to explore eHealth use among the older population as technology constantly changes and evolves and pay attention to the unintended consequences of the digitalization of health and health care. Today, it becomes increasingly difficult for patients to resist the demands of being both reflexive and empowered to act. They need to engage with this new world, where patients are required to take increasing responsibility for health-related decisions or exercise agency in this dynamic technological environment that constantly evolves.

### Conclusions

Belonging to age groups confers certain advantages and disadvantages through institutional, cultural, and interactional processes that produce and sustain age inequalities [15]. This study focused only on older eHealth users as they have crossed the chasm of being late technology adopters and overcome the structural barrier of belonging to an aged population. The structuration theory by Giddens [13,14] was a better choice to explain our findings as he posits that actors’ reflexivity is a crucial and transformative social process and their agency is only meaningful as subjects. Today, it becomes increasingly difficult for patients to resist the demands of being reflexive and empowered to act. Engaging with this new world requires patients to take increasing responsibility for health-related decisions or exercise agency. That is the attribute that separates the passive patient of 1958 from the active one today. We propose viewing eHealth use among older adults as an important factor, in which people make deliberate efforts to solve problems in their lives. Using eHealth, they express their agency, which can sometimes be challenging for late adopters as the systems are not always user-friendly and fail to provide the needed tailored services, especially in times of pandemics. Health professionals can play a vital role in this change process by encouraging older adults to use eHealth, thus eliminating socially constructed practices that ignore this change and reinforce older adults’ structural barriers.

## Appendix

Multimedia Appendix 1 eHealth definition and operationalization in studies on older adults.

## Abbreviations

HIS: health information surrogate
SRH: self-rated health

## Data Availability

The data set generated and analyzed during this study is available from the corresponding author upon reasonable request.
