# Supplementary material for: Refined Analysis of Older eHealth Users From an Agency Perspective: Quantitative Telephone Interview Study
Source: JMIR Aging. 2023 Apr 26;6:e40004. doi: 10.2196/40004 (PMC10173039; doi:10.2196/40004)
Supplement: Multimedia Appendix 1 [file aging_v6i1e40004_app1.docx]

|  |  | Definition | Participants | Questions |  | Data collection |
| --- | --- | --- | --- | --- | --- | --- |
| 1 | Choi, 2011 [32] | Health Information Technology Use (HIT) | 5,294 Americans age 65 and older | 1) looked up health information on the Internet? (2) used chat groups to learn about health topics? (3) refilled a prescription on the Internet? (4) scheduled a medical appointment on the Internet? (5) communicated with a healthcare provider by email? | Dependent | 2009 |
| 2 | Choi & Dinito, 2013 [1] | Internet use for health-related tasks | 6,680 Americans, age 65 and older | 1) Contact a medical provider. 2) handle Medicare or other health insurance matters. 3) get information about their health conditions | Dependent | 2011 |
| 3 | Gell et al., 2015 [3] | Use of internet for health-related and personal tasks | 7,065 Americans, age 65 and older | 1) went on the Internet or online to contact a medical provider, 2) handle Medicare or other health insurance matters, and 3) get information about one’s health conditions. | Dependent | 2011 |
| 4 | Tennant et al, 2015 [35] | Use of Web 2.0 for health information | 283 Americans, age 50 and older | (1) participated in a Web-based-support group, (2) used a social networking site like Facebook/Twitter/ LinkedIn, (3) wrote in a Web-based diary or blog. | Dependent | 2013 |
| 5 | Bujnowska-Fedak & Mastalerz-Migas, 2015 [37] | Usage of Medical Internet and e-Health Services | 242 Polish, age 60 and older | 1) Obtain information about health or illness, 2) approach family physician, specialist, or other health professionals over the Internet, 3) search the doctor’s own website, 4) get access to one’s own medical records, 5) schedule or change appointments online | Dependent | 2012 |
| 6 | Shahrabani & Mizrachi, 2016 [36] | online health-related actions | 703 Israeli, age 45 and older | 1) Frequency of using Health Maintenance Organization website for administrative information, 2) consult with physician online, 3) obtain medical information from a forum | Dependent | 2014 |
| 7 | Parida et al., 2016 [33] | Use of social media for health-related activities | 610 Swedish, age 59 and older | 1) getting health information, (2) starting or joining a health-related group, (3) following friends’ personal health experiences and health updates, (4) raising money or drawing attention to a health-related issue or cause, and (5) remembering or memorializing others who suffered from a certain health condition | Dependent | NA |
| 8 | Hong & Cho, 2017 [5] | health-related Internet use (HRIU) | 2,150, 2,503, and 1950 Americans, age 55 and older | (1) seek health information for self or others, (2) buy medicine or vitamins, (3) connect with people with similar health problems, and (4) communicate with doctors in the past 12 months | Dependent | 2003, 2005, 2011 respectively |
| 9 | Levine, Lipsitz & Linder, 2018 [34] | Digital health technology | 4,037 Americans, age 65 and older | 1) use of the Internet to research health conditions, 2) contact clinicians, 3) fill prescriptions, and 4) address insurance matters | Dependent | 2011-2014 |
| 10 | Shim et al., 2018. [6] | Web-based health information | 991 Americans, age 52 and older | 1) websites use for finding medical and health information? | Independent | 2012, 2014 |
| 11 | Seckin et al., 2018. [4] | Online health information seeking | 499 Americans, age 40 and older  194 Americans, age 60 and older | 1) frequency of health or medical information seeking on the Internet, 2) consulting the Internet *before* visiting a healthcare provider, 3) consulting the Internet *after* visiting a healthcare provider. 4) do you discuss the information you obtained from the internet with a healthcare provider? | Independent | NA |
| 12 | Seckin et al., 2019. [31] | e-Health information seeking  e-Health information consumerism | 499 Americans, age 40 and older  194 Americans, age 60 and older | 1) frequency of health or medical information seeking on the Internet, 2) consulting the Internet *before* visiting a healthcare provider, 3) consulting the Internet *after* visiting a healthcare provider. 4) do you discuss the information you obtained from the internet with a healthcare provider? 5)Do you ask a health care provider for advice about where to find credible health or medical information on the internet?  1) Do you seek information on the internet to self-diagnose? 2) Do you use information from the internet to identify appropriate treatments for yourself? 3) Do you request medication from a healthcare provider based on the Internet information? 4) Do you request medical examination from a healthcare provider based on the Internet information? 5) Do you request treatment from a healthcare provider based on the Internet information? 6) Do you purchase medication based on the Internet information? 7) Do you treat a health issue based on the Internet information? 8) Do you use information from the Internet to make treatment decisions? | Independent | NA |
| 13 | Weber et al., 2020 [38] | Online information seeking | 701 Germans, age 60 and older | 1) frequency of health or medical information seeking on the Internet, 2) frequency of health or medical information seeking on apps | Dependent | NA |

Multimedia appendix 1. eHealth definition and operationalization in studies on older adults.

Table 1: e-Health definition and operationalization in studies among older adults
